# Supplementary material for: Gastric Neuroendocrine Tumors (g-NETs): A Systematic Review of the Management and Outcomes of Type 3 g-NETs
Source: Cancers (Basel). 2023 Apr 8;15(8):2202. doi: 10.3390/cancers15082202 (PMC10137004; doi:10.3390/cancers15082202)
Supplement: Supplementary file 1 [file cancers-15-02202-s001.zip › cancers-2275558-supplementary.pdf]

Table S1 – Search strings used for performing the systematic review of the literature.

PUBMED and MEDLINE	EMBASE
((((((("management"[Title/Abstract] OR "outcome"[Title/Abstract] OR "treatment"[Title/Abstract] OR "survival"[Title/Abstract] OR "surviving"[Title/Abstract] OR "outcome"[All Fields] OR "outcomes"[All Fields] OR "treatment"[All Fields] OR "treatments"[All Fields] OR "survival"[All Fields] OR "surviv*"[All Fields] OR "surviving"[All Fields]) AND ("neuroendocrine neoplasms"[Title/Abstract] OR "neuroendocrine neoplasm"[Title/Abstract] OR "neuroendocrine tumours"[Title/Abstract] OR "neuroendocrine tumors"[Title/Abstract] OR "neuroendocrine cancers"[Title/Abstract] OR "neuroendocrine cancer"[Title/Abstract] OR "NET"[Title/Abstract] OR "NEN"[Title/Abstract] OR "NETs"[Title/Abstract] OR "NENs"[Title/Abstract] OR "neuroendocrine neoplasms"[All Fields] OR "neuroendocrine neoplasm"[All Fields] OR "neuroendocrine tumours"[All Fields] OR "neuroendocrine tumors"[All Fields] OR "neuroendocrine cancers"[All Fields] OR "neuroendocrine cancer"[All Fields] OR "NET"[All Fields] OR "NEN"[All Fields] OR "NETs"[All Fields])) OR "NENs"[All Fields]) AND ("gastric"[Title/Abstract] OR "stomach"[Title/Abstract] OR "gastr*"[All Fields] OR "abdom*"[All Fields])) OR "stomach"[All Fields]) AND ("type 3"[Title/Abstract] OR "type III"[Title/Abstract] OR "type III"[All Fields] OR "type 3"[All Fields]) AND ("neurosecretory systems"[MeSH Terms] OR "stomach neoplasms"[MeSH Terms] OR "stomach neoplasms"[MeSH Terms] OR "stomach neoplasms"[MeSH Terms] OR "stomach neoplasms"[MeSH Terms] OR "neuroendocrine tumors"[MeSH Terms] OR "neuroendocrine tumors"[MeSH Terms])) NOT "review"[Publication Type]) NOT "adenocarcinoma*"[MeSH Terms]	('neuroendocrine tumor'/exp OR 'neuroendocrine tumor':ti,ab,kw OR 'neuroendocrine tumors':ti,ab,kw OR 'neuroendocrine tumour':ti,ab,kw OR 'neuroendocrine tumours':ti,ab,kw) AND ('type iii':ti,ab OR 'type 3':ti,ab) AND ('management'/exp OR 'treatment outcome'/exp OR 'health care outcome and process assessment' OR 'healthcare outcome and process assessment' OR 'medical futility' OR 'outcome and process assessment (health care)' OR 'outcome and process assessment, health care' OR 'outcome management' OR 'patient outcome' OR 'therapeutic outcome' OR 'therapy outcome' OR 'treatment outcome')
